# Supplementary material for: In situ cryo-electron tomography reveals the asymmetric architecture of mammalian sperm axonemes
Source: Nat Struct Mol Biol. 2023 Jan 2;30(3):360–9. doi: 10.1038/s41594-022-00861-0 (PMC10023559; doi:10.1038/s41594-022-00861-0)
Supplement: Supplementary file 1 — Supplementary Figs. 1 and 2 and Table 1 [file 41594_2022_861_MOESM1_ESM.pdf]

nature structural & molecular biology

Article https://doi.org/10.1038/s41594-022-00861-0

In situ cryo-electron tomography reveals the asymmetric architecture of mammalian sperm axonemes

In the format provided by the authors and unedited

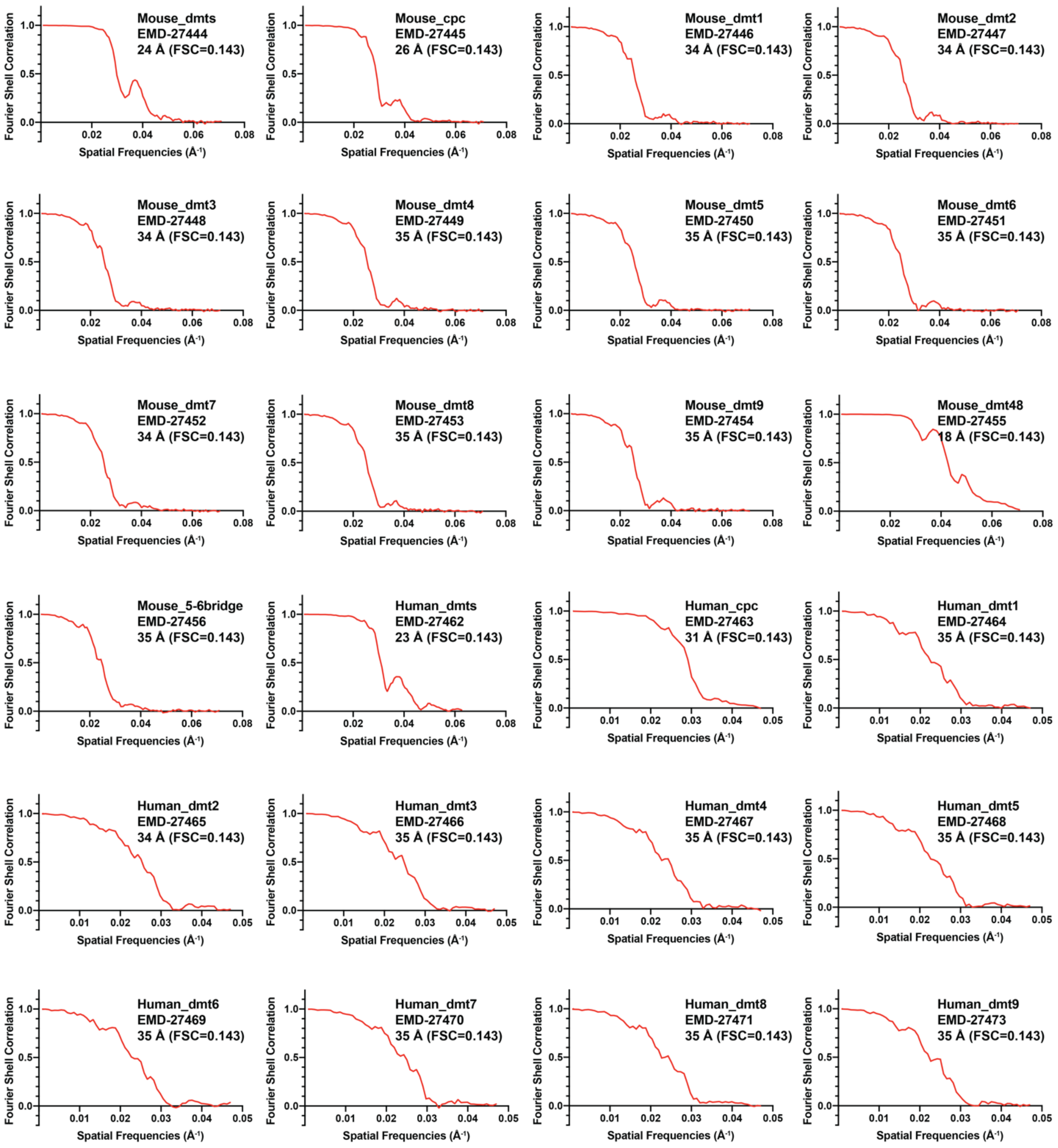

Supplementary Data Figure 1. Gold-standard Fourier shell correlation (FSC) curves of subvolume averages. The FSC curves are labeled with the names of the structures, the EMDB deposition numbers, and the resolution estimate based on FSC = 0.143 standard.

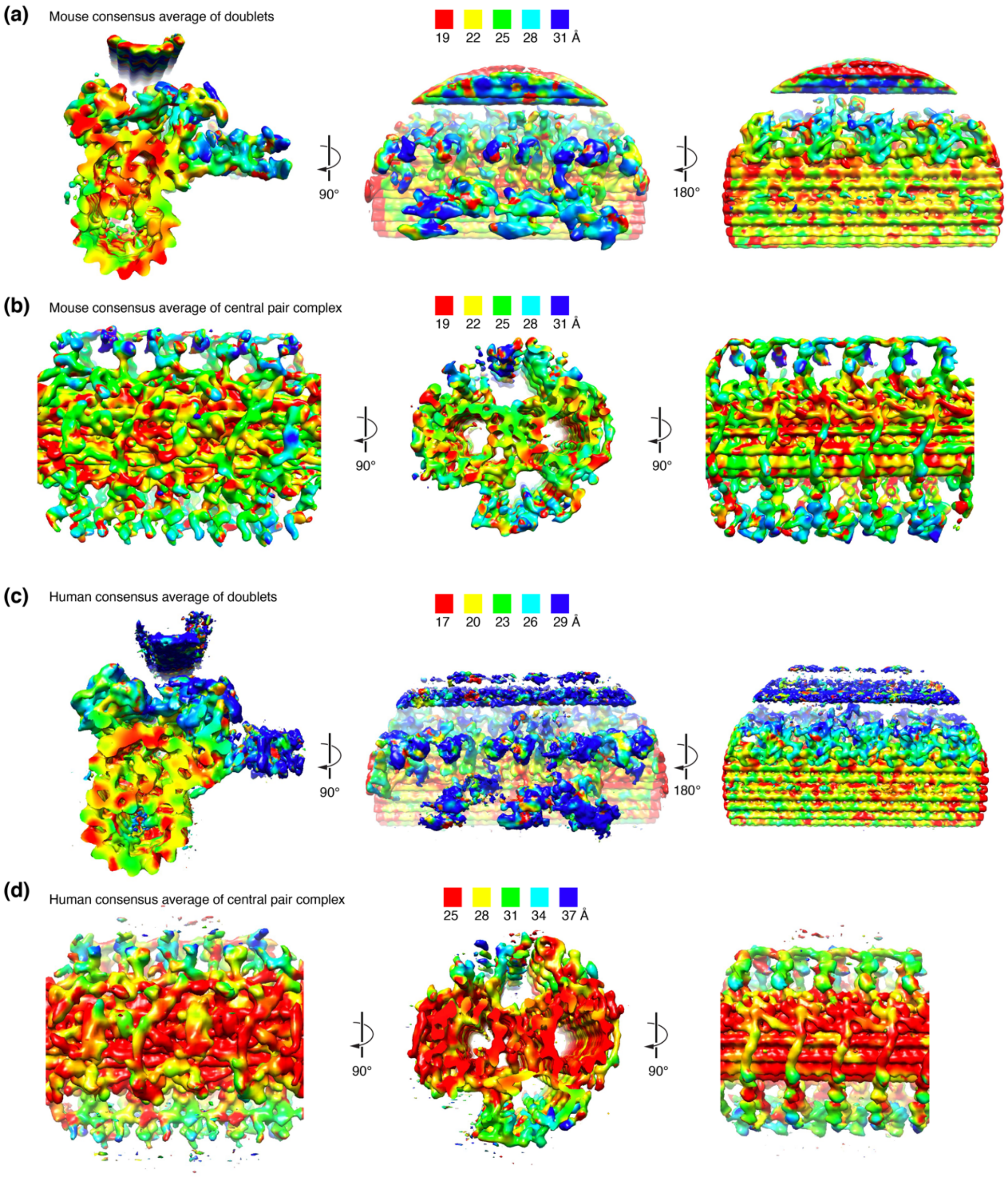

Supplementary Data Figure 2. Local resolution maps of the consensus averages of mouse and human sperm. The local resolution maps were generated using blocres from the Bsoft package. These local-resolution maps illustrate relative differences in resolution across the maps but the absolute values may not be exact. In general, structural features near the microtubules are better resolved than those that are further away.

Supplementary Table S1. Annotated list of protein candidates from Mass Spectrometry analyses of KSCN extraction of bovine sperm[42].

Gene symbol	Assignment	Reference
HSPA1L	Central pair complex	Han et al, *Nat. Strut. Mol. Biol.* 2022. Gui et al, Nat. Strut. Mol. Biol. 2022.
HYDIN	Central pair complex	Han et al, *Nat. Strut. Mol. Biol.* 2022. Gui et al, Nat. Strut. Mol. Biol. 2022.
KIF9	Central pair complex	Han et al, *Nat. Strut. Mol. Biol.* 2022. Gui et al, Nat. Strut. Mol. Biol. 2022.
LRGUK	Central pair complex	Han et al, *Nat. Strut. Mol. Biol.* 2022. Gui et al, Nat. Strut. Mol. Biol. 2022.
MYCBP	Central pair complex	Han et al, *Nat. Strut. Mol. Biol.* 2022. Gui et al, Nat. Strut. Mol. Biol. 2022.
PPP1CC	Central pair complex	Han et al, *Nat. Strut. Mol. Biol.* 2022. Gui et al, Nat. Strut. Mol. Biol. 2022.
SPAG16	Central pair complex	Han et al, *Nat. Strut. Mol. Biol.* 2022. Gui et al, Nat. Strut. Mol. Biol. 2022.
SPAG17	Central pair complex	Han et al, *Nat. Strut. Mol. Biol.* 2022. Gui et al, Nat. Strut. Mol. Biol. 2022.
SPAG6	Central pair complex	Han et al, *Nat. Strut. Mol. Biol.* 2022. Gui et al, Nat. Strut. Mol. Biol. 2022.
SPATA4	Central pair complex	Han et al, *Nat. Strut. Mol. Biol.* 2022. Gui et al, Nat. Strut. Mol. Biol. 2022.
SPEF2	Central pair complex	Han et al, *Nat. Strut. Mol. Biol.* 2022. Gui et al, Nat. Strut. Mol. Biol. 2022.
WDR93	Central pair complex	Han et al, *Nat. Strut. Mol. Biol.* 2022. Gui et al, Nat. Strut. Mol. Biol. 2022.
CCDC11	MIP	Gui et al, *Cell* 2021.
CCDC173	MIP	Gui et al, *Cell* 2021.
CCDC19	MIP	Gui et al, *Cell* 2021.
EFCAB6	MIP	Gui et al, Cell 2021.
EFHB	MIP	Gui et al, *Cell* 2021.
EFHC1	MIP	Gui et al, *Cell* 2021.
EFHC2	MIP	Gui et al, *Cell* 2021.
ENKUR	MIP	Gui et al, *Cell* 2021.
MNS1	MIP	Gui et al, *Cell* 2021.
NME7	MIP	Gui et al, *Cell* 2021.
PACRG	MIP	Gui et al, *Cell* 2021.
RIBC2	MIP	Gui et al, *Cell* 2021.
SPAG8	MIP	Gui et al, *Cell* 2021.
TEKT1	MIP	Gui et al, *Cell* 2021.
TEKT2	MIP	Gui et al, *Cell* 2021.
TEKT3	MIP	Gui et al, *Cell* 2021.
TEKT4	MIP	Gui et al, *Cell* 2021.
TEKT5	MIP (likely)	
GAS8	N-DRC	Jeanson et al, *Hum. Mutat.* 2016.
IQCA1	N-DRC	Homolog found in *Chlamydomonas*: Bower et al., *Mol. Biol. Cell* 2013.
LRRC48	N-DRC	Homolog found in *Chlamydomonas*: Bower et al., *Mol. Biol. Cell* 2013.
CCDC135	N-DRC	Homolog found in *Chlamydomonas*: Bower et al., *Mol. Biol. Cell* 2013.

CCDC65	N-DRC	Homolog found in *Chlamydomonas*: Bower et al., *Mol. Biol. Cell* 2013.
IQCD	N-DRC	Homolog found in *Chlamydomonas*: Bower et al., *Mol. Biol. Cell* 2013.
IQCG	N-DRC	Homolog found in *Chlamydomonas*: Bower et al., *Mol. Biol. Cell* 2013.
TCTE1	N-DRC	Homolog found in *Chlamydomonas*: Bower et al., *Mol. Biol. Cell* 2013.
ARMC4	ODA docking complex	Gui et al, *Cell* 2021.
CCDC114	ODA docking complex	Gui et al, *Cell* 2021.
CCDC151	ODA docking complex	Gui et al, *Cell* 2021.
TTC25	ODA docking complex	Gui et al, *Cell* 2021.
CYB5D1	Radial spoke	Homolog found in *Chlamydomonas*: Gui et al, *Nat. Strut. Mol. Biol.* 2021.
DNAJB13	Radial spoke	Homolog found in *Chlamydomonas*: Gui et al, *Nat. Strut. Mol. Biol.* 2021.
DYNLL1	Radial spoke	Homolog found in *Chlamydomonas*: Gui et al, *Nat. Strut. Mol. Biol.* 2021.
IQUB	Radial spoke	Homolog found in *Chlamydomonas*: Gui et al, *Nat. Strut. Mol. Biol.* 2021.
LRRC34	Radial spoke	Homolog found in *Chlamydomonas*: Gui et al, *Nat. Strut. Mol. Biol.* 2021.
MAATS1	Radial spoke	Homolog found in *Chlamydomonas*: Gui et al, *Nat. Strut. Mol. Biol.* 2021.
NME5	Radial spoke	Homolog found in *Chlamydomonas*: Gui et al, *Nat. Strut. Mol. Biol.* 2021.
PPIL6	Radial spoke	Homolog found in *Chlamydomonas*: Gui et al, *Nat. Strut. Mol. Biol.* 2021.
RSPH1	Radial spoke	Homolog found in *Chlamydomonas*: Gui et al, *Nat. Strut. Mol. Biol.* 2021.
RSPH3	Radial spoke	Homolog found in *Chlamydomonas*: Gui et al, *Nat. Strut. Mol. Biol.* 2021.
RSPH6A	Radial spoke	Homolog found in *Chlamydomonas*: Gui et al, *Nat. Strut. Mol. Biol.* 2021.
RSPH9	Radial spoke	Homolog found in *Chlamydomonas*: Gui et al, *Nat. Strut. Mol. Biol.* 2021.
TUBA1A	Tubulin	
TUBA1D	Tubulin	
TUBA3E	Tubulin	
TUBA8	Tubulin	
TUBB2B	Tubulin	
TUBB2C	Tubulin	
TUBB3	Tubulin	
DNAH1	Dynein heavy chain	
DNAH10	Dynein heavy chain	
DNAH17	Dynein heavy chain	
DNAH2	Dynein heavy chain	
DNAH3	Dynein heavy chain	
DNAH5	Dynein heavy chain	
DNAH6	Dynein heavy chain	
DNAH7	Dynein heavy chain	
DNAH8	Dynein heavy chain	
SEPT10		
SEPT12		
SEPT4		

SEPT7		
ACADVL		
ACE		
ACRV1		
ACSS1		
ACTB		
ACTL7A		
ACTRT2		
ADGB		
ADRBK2		
AK1		
AK7		
AK8		
AK9		
AKAP3		
AKAP4		
AKR1B1		
AKR1B10		
ALDOA		
ANKRD5		
ARMC12		
ARMC3		
ASRGL1		
ATP5A1		
ATP5B		
ATP5O		
BSP1		
BTBD16		
C10orf82		
C11H2orf39		
C11H9ORF116		
C11H9ORF9		
C12H13ORF26		
C12orf55		
C13H20ORF85		
C16orf80		
C16orf93		
C16orf96		
C17orf47		

C19orf45		
C1orf192		
C1orf194		
C1orf228		
C20orf26		
C21H15ORF26		
C29H11orf66		
C2orf70		
C3H1ORF92		
C3orf30		
C3orf84		
C4orf47		
C7orf31		
C7orf62		
C7orf63		
C7orf72		
C9H6orf163		
C9H6ORF165		
C9orf117		
C9orf171		
CABYR		
CALM		
CALR		
CAMK4		
CAPZA3		
CAPZB		
CASC1		
CCDC105		
CCDC108		
CCDC113		
CCDC116		
CCDC136		
CCDC146		
CCDC147		
CCDC176		
CCDC27		
CCDC38		
CCDC39		
CCDC40		

CCDC42		
CCDC63		
CCDC81		
CCDC87		
CCDC96		
CCIN		
CCT2		
CCT3		
CCT4		
CCT5		
CCT6A		
CCT7		
CCT8		
CCT8L2		
CES7		
CETN1		
CLGN		
CLTC		
CLU		
COX6B2		
CSE1L		
CSNK2A1		
CSNK2A2		
CSNK2B		
CXorf22		
CXorf30		
CXorf30		
CXorf65		
CYB5R1		
CYLC1		
CYLC2		
DKFZp686E0752		
DLAT		
DLEC1		
DLST		
DNAI1		
DNAI2		
DNAJB6		
DNAL1		

DNAL4		
DNALI1		
DNHD1		
DPY30		
DUSP18		
DYDC1		
DYNLL2		
DYNLRB2		
DYNLT1		
EEF1A1		
EEF1G		
EFCAB1		
EFCAB10		
EFCAB2		
EFCAB3		
EFCAB5		
EIF3F		
ELP2P		
ENO1		
ENO4		
FAM154A		
FAM161A		
FAM166A		
FAM183B		
FNDC8		
FSCB		
FSCN3		
FSIP2		
FSIP2		
FTL		
GALK1		
GAPDH		
GAPDHS		
GK		
GK2		
GLB1L2		
GLUL		
GPX4		
GPX5		

GSN		
GSTM3		
GSTO2		
GSTT3		
H2AFZ		
HIST1H4A		
HIST2H2BF		
HK1		
HNRNPK		
HSP90AA1		
HSP90B1		
HSPA2		
HSPA5		
HSPB9		
HYOU1		
IL4I1		
IQCA1P1		
IQCF1		
IQCK		
IRGC		
ISYNA1		
KIAA1683		
KIAA1984		
LDHA		
LDHC		
LRP2BP		
LRRC18		
LRRC23		
LRRC37A		
LRRC43		
LRRC74		
LRRD1		
LRRIQ4		
LUZP2		
LZTFL1		
MAN2B2		
MATR3		
MDH1		
MDH1B		

MDH2		
MENT		
MLF1		
MORN3		
MTCH2		
MYCBPAP		
NT5C1B		
NUP210L		
OAZ3		
ODF1		
ODF2		
ODF3		
PCMT1		
PDHB		
PDIA3		
PFN3		
PGAM2		
PGK2		
PKM2		
PLG		
POC1A		
PPIA		
PPP1CA		
PPP2R1A		
PPP2R5A		
PRDX5		
PRKACA		
PRKACB		
PRKAR1A		
PRKAR2A		
QRICH2		
RAB2A		
RAN		
RANBP6		
RGS22		
ROPN1		
ROPN1L		
RSPH10B		
RTDR1		

SAMD15		
SCCPDH		
SGK071		
SH3GLB1		
SLC25A31		
SMRP1		
SPA17		
SPATA18		
SPATA20		
SPATA31A4		
SPATA32		
SPATC1		
SPERT		
STYXL1		
TCEB2		
TCP1		
TCP11		
TCTE3		
TEPP		
TEX33		
TEX43		
THEG		
TPI1		
TSC21		
TSGA10		
TSKS		
TSNAXIP1		
TSSK1B		
TSSK6		
TTC18		
TTC29		
TTC40		
TTN		
TXNDC3		
TXNDC8		
UBA52		
UBXN11		
UQCRC1		
VCP		

VDAC2		
VDAC3		
WDR16		
WDR51B		
WDR52		
WDR63		
WDR64		
WDR65		
WDR66		
WDR78		
WDR96		
YWHAE		
YWHAZ		
ZAN		
ZMYND12		
ZPBP		

Supplementary Movie 1. The structure of the radial spoke barrel.

Supplementary Movie 2. Multi-body refinement of radial spokes from doublet 6 and the nearby central pair protrusions. The movie contains 10 frames and each frame represents an average of 1/10 of the total populations distributed along the first component of the Principal Component Analysis. The different frames indicate that the radial spokes and the central pair complex can come together at different longitudinal offsets. The interface between doublet 6 and the central pair protrusion 1b is used as an example here.
