# Supplementary material for: In situ cryo-electron tomography reveals the asymmetric architecture of mammalian sperm axonemes
Source: Nat Struct Mol Biol. 2023 Jan 2;30(3):360–9. doi: 10.1038/s41594-022-00861-0 (PMC10023559; doi:10.1038/s41594-022-00861-0)
Supplement: Supplementary file 2 — Reporting Summary [file 41594_2022_861_MOESM2_ESM.pdf]

nature portfolio

Corresponding author(s): Ronald D. Vale; David A. Agard

Last updated by author(s): Sep 21, 2022

Reporting Summary

Nature Portfolio wishes to improve the reproducibility of the work that we publish. This form provides structure for consistency and transparency in reporting. For further information on Nature Portfolio policies, see our Editorial Policies and the Editorial Policy Checklist.

Statistics

For all statistical analyses, confirm that the following items are present in the figure legend, table legend, main text, or Methods section.

n/a	Confirmed	
☐	☒	The exact sample size (*n*) for each experimental group/condition, given as a discrete number and unit of measurement
☐	☒	A statement on whether measurements were taken from distinct samples or whether the same sample was measured repeatedly
☒	☐	The statistical test(s) used AND whether they are one- or two-sided<br>*Only common tests should be described solely by name; describe more complex techniques in the Methods section.*
☒	☐	A description of all covariates tested
☒	☐	A description of any assumptions or corrections, such as tests of normality and adjustment for multiple comparisons
☒	☐	A full description of the statistical parameters including central tendency (e.g. means) or other basic estimates (e.g. regression coefficient) AND variation (e.g. standard deviation) or associated estimates of uncertainty (e.g. confidence intervals)
☒	☐	For null hypothesis testing, the test statistic (e.g. *F*, *t*, *r*) with confidence intervals, effect sizes, degrees of freedom and *P* value noted<br>*Give P values as exact values whenever suitable.*
☒	☐	For Bayesian analysis, information on the choice of priors and Markov chain Monte Carlo settings
☒	☐	For hierarchical and complex designs, identification of the appropriate level for tests and full reporting of outcomes
☒	☐	Estimates of effect sizes (e.g. Cohen's *d*, Pearson's *r*), indicating how they were calculated

Our web collection on statistics for biologists contains articles on many of the points above.

Software and code

Policy information about availability of computer code

Data collection	SerialEM3.8.4
Data analysis	The EM software used: RELION v1.4, v3.0.6, Motioncorr v2; Structural visualization: Chimera 8.6.1, ChimeraX 1.1, IMOD 4.9.13

For manuscripts utilizing custom algorithms or software that are central to the research but not yet described in published literature, software must be made available to editors and reviewers. We strongly encourage code deposition in a community repository (e.g. GitHub). See the Nature Portfolio guidelines for submitting code & software for further information.

Data

Policy information about availability of data

All manuscripts must include a data availability statement. This statement should provide the following information, where applicable:

- Accession codes, unique identifiers, or web links for publicly available datasets
- A description of any restrictions on data availability
- For clinical datasets or third party data, please ensure that the statement adheres to our policy

The maps of the following structures are available in the Electron Microscopy Data Bank: EMD-27444, consensus average of 96 nm-repeating structure of mouse doublets; EMD-27445, 32 nm-repeating structure of central pair complex of mouse sperm; EMD-27446, mouse doublet 1; EMD-27447, mouse doublet 2; EMD-27448, mouse doublet 3; EMD-27449, mouse doublet 4; EMD-27450, mouse doublet 5; EMD-27451, mouse doublet 6; EMD-27452, mouse doublet 7; EMD-27453, mouse doublet 8; EMD-27454, mouse doublet 9; EMD-27455, 48 nm-repeating structure of doublet microtubule of mouse sperm; EMD-27456, 5-6 bridge of mouse sperm; EMD-27462, consensus average of 96 nm-repeating structure of human doublets; EMD-27463, 32 nm-repeating structure of central pair complex of human sperm; EMD-27464, human doublet 1; EMD-27465, human doublet 2; EMD-27466, human doublet 3; EMD-27467, human doublet 4;

EMD-27468, human doublet 5; EMD-27469, human doublet 6; EMD-27470, human doublet 7; EMD-27471, human doublet 8; EMD-27473, human doublet 9. The 69 raw tilt series of mouse sperm lamellae and the corresponding tilt angle files are available in the EMPIAR database (EMPIAR-11221).

Field-specific reporting

Please select the one below that is the best fit for your research. If you are not sure, read the appropriate sections before making your selection.

☒ Life sciences ☐ Behavioural & social sciences ☐ Ecological, evolutionary & environmental sciences

For a reference copy of the document with all sections, see nature.com/documents/nr-reporting-summary-flat.pdf

Life sciences study design

All studies must disclose on these points even when the disclosure is negative.

Sample size	The mouse sperm cryoEM datasets were collected in 6 independently prepared samples and no statistical methods were used to predetermined sample size. The data were processed in batches and the biological structures were observed to be highly consistent between batches. Each EM grids usually contains millions of sperm cells and we randomly sampled cells for cryoET imaging. In total, 69 sperm/regions were stochastically sampled and all subtomogram averages were performed with N>700 particles from > 50 axoneme segments. We determined this to be sufficient owing to the consistency of the averages from three batches of datasets. Human sperm from a volunteer were collected and the cells were were normozoospermic with typical motility and morphologies.
Data exclusions	Through 3D classification procedures, we discarded "duplicated particles" that result from initial overpicking of particles based on their positions in the raw data. The procedure ensures that we only counted one subtomogram once in our data and this is standard and established in the cryoEM field.
Replication	The data were independently processed in 3 batches and similar results were obtained (with varied levels of noise depending on the size of the batch). All data were combined and used to make the figures.
Randomization	For cryoET imaging, there are millions of sperm cells on each grid and individual sperm were selected at random without bias toward sample location, size or fields under the microscope. For the data processing, we selected particles corresponding to specific cellular structures, which is standard in the cryoEM field. The gold-standard Fourier shell correlation was calculated from two independently refined half-maps, calculated based on two randomly selected halves of data.
Blinding	At the begining of study, we assume that we do not know the true mammalian sperm structures. Initial reference of the equivalent structures from Tetrahymena cilia and sea urchin sperm were low-pass filtered to 60 angstrom to eliminate the reference bias. Still, sperm-specific densities were found in the final reconstruction at ~25 angstrom resolution (higher than the reference), suggesting that these signals came from the actual data but not the initial reference.

Reporting for specific materials, systems and methods

We require information from authors about some types of materials, experimental systems and methods used in many studies. Here, indicate whether each material, system or method listed is relevant to your study. If you are not sure if a list item applies to your research, read the appropriate section before selecting a response.

Materials & experimental systems

n/a	Involved in the study	
☒	☐	Antibodies
☒	☐	Eukaryotic cell lines
☒	☐	Palaeontology and archaeology
☐	☒	Animals and other organisms
☐	☒	Human research participants
☒	☐	Clinical data
☒	☐	Dual use research of concern

Methods

n/a	Involved in the study	
☒	☐	ChIP-seq
☒	☐	Flow cytometry
☒	☐	MRI-based neuroimaging

Animals and other organisms

Policy information about studies involving animals; ARRIVE guidelines recommended for reporting animal research

Laboratory animals	C57Bl/6J mice were housed on a free-standing, individually ventilated (~60 air changes hourly) rack (Allentown Inc, Allentown, NJ). The holding room was ventilated with 100% outside filtered air with at 15 to 20 air changes hourly. Each ventilated cage (Allentown) was provided with corncob bedding (Shepard Specialty Papers, Milford, NJ), at least 8g of nesting material (Bed-r'Nest, The Andersons, Maumee, OH), and red Mouse Tunnel (Bio-Serv, Flemington, NJ). Mice were maintained on a 12:12-h light:dark cycle. The holding room temperature was maintained at 21±1°C with a relative humidity of 30% to 70%. Irradiated rodent laboratory chow (LabDiet 5053) was provided ad libitum and chlorinated (1-3ppm) RO water was provided without restriction.

Wild animals	No wild animals were used in the study.
Field-collected samples	No field collected samples were used in the study.
Ethics oversight	All mice were cared for in compliance with the Guide for the Care and Use of Laboratory Animals. All experiments were approved by the Janelia Research Campus' (JRC) IACUC. JRC is an AAALAC-accredited institution.

Note that full information on the approval of the study protocol must also be provided in the manuscript.

Human research participants

Policy information about studies involving human research participants

Population characteristics	A man aged 25-39 years old were recruited and consented to participate in this study. Freshly ejaculated semen samples were obtained by masturbation. All processed samples were normozoospermic with a cell count of at least 30 million sperm cells per milliliter.
Recruitment	Healthy volunteers were recruited randomly and provided informed consent. All volunteers showed normal semen analysis at the time of sample collection.
Ethics oversight	The experimental procedures utilizing human-derived sperm samples were approved by the Committee on Human Research at the University of California, Berkeley, IRB protocol number 2013-06-5395.

Note that full information on the approval of the study protocol must also be provided in the manuscript.
