# Supplementary material for: In situ cryo-electron tomography reveals the asymmetric architecture of mammalian sperm axonemes
Source: Nat Struct Mol Biol. 2023 Jan 2;30(3):360–9. doi: 10.1038/s41594-022-00861-0 (PMC10023559; doi:10.1038/s41594-022-00861-0)
Supplement: Supplementary file 6 — Supplementary Code [file 41594_2022_861_MOESM6_ESM.pdf]

Supplementary Code 1

Script to remap coordinates of subvolumes in three dimensions

```
# Take input of x,y,z coordinates (space separated) and output a Chimera markers file (.cmm suffix).
#
# Example use
#
#   python3 make_markers.py tomo0112.coords > tomo0112.cmm
#
# Here's documentation on the Chimera marker file format.
#
#
https://www.cgl.ucsf.edu/chimera/docs/ContributedSoftware/volumepathtracer/volumepathtracer.html#ma
rkerfiles
#

voxel_size = 1.0        # Scale input x,y,z coordinates to get Angstroms
radius = 10             # Radius of marker spheres in Angstroms.

from sys import argv, stdout
input_filename = argv[1]
input_lines = open(input_filename).readlines()
lines = ['<marker_set name="%s">' % input_filename]
for i, line in enumerate(input_lines):
    x,y,z = [voxel_size * float(x) for x in line.split()]
    lines.append('<marker x="%.2f" y="%.2f" z="%.2f" radius="%.2f" id="%d" />'
                 % (x,y,z,radius,i+1))
lines.append('</marker_set>')

stdout.write('\n'.join(lines))
```
